# A Material Approach to Endangered Species Conservation: Characterization and 3D Imaging of Ballistic Damage in the Casques of Helmeted Hornbill (*Rhinoplax vigil*)

**DOI:** 10.1093/iob/obag001

**Published:** 2026-01-19

**Authors:** T Y Chan, V Kamska, M Schindler, S Xie, T F Kong, R Hu, M N Dean

**Affiliations:** Department of Infectious Diseases and Public Health, Jockey Club College of Veterinary Medicine and Life Sciences, City University of Hong Kong, 83 Tat Chee Avenue, Kowloon, Hong Kong; Department of Infectious Diseases and Public Health, Jockey Club College of Veterinary Medicine and Life Sciences, City University of Hong Kong, 83 Tat Chee Avenue, Kowloon, Hong Kong; Department of Infectious Diseases and Public Health, Jockey Club College of Veterinary Medicine and Life Sciences, City University of Hong Kong, 83 Tat Chee Avenue, Kowloon, Hong Kong; Mandai Wildlife Group, 80 Mandai Lake Road 729826, Singapore; Department of Industrial and Systems Engineering, The Hong Kong Polytechnic University, Hung Hom, Hong Kong; Department of Industrial and Systems Engineering, The Hong Kong Polytechnic University, Hung Hom, Hong Kong; Department of Infectious Diseases and Public Health, Jockey Club College of Veterinary Medicine and Life Sciences, City University of Hong Kong, 83 Tat Chee Avenue, Kowloon, Hong Kong; Centre for Nature-Inspired Engineering, City University of Hong Kong, 83 Tat Chee Avenue, Kowloon Tong, Hong Kong; Department of Biomaterials, Max Planck Institute of Colloids & Interfaces, Am Muehlenberg 1, 14484 Potsdam, Germany

**Keywords:** non-destructive forensics, microcomputed tomography, trabeculae, bone callus, human-wildlife interactions

## Abstract

The helmeted hornbill (*Rhinoplax vigil*), a critically endangered bird prized for the distinctive helmet-like “casque” capping its beak, faces threats from poaching and habitat loss. This study applies biomaterials/imaging techniques to wildlife forensics, using micro-computed tomography to forensically analyse bullet-induced damage in a unique collection of helmeted hornbill skull specimens, confiscated by the Agriculture, Fisheries and Conservation Department (AFCD) of Hong Kong. The findings reveal diverse healing responses, bullet characteristics and indications of the poaching strategies used. For example, specimen scans—showing various bullets embedded in skull tissue—suggest that poachers may use the conspicuous casque as a target when shooting upward from the ground. Additionally, our observations of casque anatomy exhibit two different types of tissue repair, arguing that several of the individuals lived long after being shot. The study highlights the value of confiscated specimens for understanding life history factors and poaching impacts in rare species, vital for developing tailored conservation strategies and advancing knowledge of the biology of elusive wildlife.

## German

Der vom Aussterben bedrohte Schildhornvogel (*Rhinoplax vigil*) zeichnet sich durch sein charakteristisches „Schnabelhorn“, welches den Schnabel ummantelt aus und ist dadurch besonders durch Wilderei, aber auch durch Verlust seines Habitats bedroht. In dieser Studie nutzen wir Biomaterialien- und Visualisierungmethoden im Bereich der Wildtierforensik und verwenden Mikro-Computertomographie für die forensische Bewertung von durch Geschosse verursachte Schäden an den Schädeln von Schildhornvögeln. Zurückgegriffen wird dabei auf eine besondere Sammlung von gewilderten Schädeln, die vom Amt für Landwirtschaft, Fischerei und Naturschutz (AFCD) in Hongkong beschlagnahmt wurden. Unsere Ergebnisse zeigen unterschiedliche Heilungsreaktionen, Merkmale der genutzten Projektile und weitere Hinweise auf Wilderei-Strategien. Beispielsweise deuten die Scans der Exemplare, die verschiedene im Schädelgewebe eingebettete Geschossfragmente vorweisen, darauf hin, dass Wilderer das auffällige Schnabelhorn als Ziel anvisieren, wenn sie vom Boden aus nach oben auf die Tiere schießen. Zudem zeigen unsere anatomischen Auswertungen der Wundheilung des Schnabelhorns zwei unterschiedliche Arten der Gewebereparatur, was darauf hindeutet, dass einige der Tiere nach dem erfolgten Beschuss noch länger gelebt haben. Die Studie betont die Bedeutung von beschlagnahmten Tierprodukten für die Naturkunde und die Auswirkung von Wilderei auf seltene Arten, wodurch entscheidende ökologische Kenntnisse gewonnen und entsprechende Schutzstrategien konzipiert werden können.

## Ukrainian

Шоломоносний птах-носоріг (*Rhinoplax vigil*), критично зникаючий вид птахів, цінований за характерний «шолом», що вкриває його дзьоб, перебуває під загрозою через браконьєрство та втрату середовища існування. У цьому дослідженні методи біоматеріалознавства та візуалізації застосовуються у судово-біологічному аналізі дикої природи: за допомогою мікрокомп’ютерної томографії проведено судово-експертне дослідження пошкоджень, спричинених кулями, в унікальній колекції черепів шоломоносного птаха-носорога, конфіскованих Департаментом Сільського Господарства, Рибальства та Охорони Природи Гонконгу (AFCD). Отримані результати виявляють різноманітні реакції загоєння тканин, характеристики куль, а також ознаки застосовуваних браконьєрами стратегій полювання. Зокрема, томографічні зображення зразків—що демонструють різні кулі, вбудовані в тканини черепа—свідчать про те, що браконьєри могли використовувати добре помітний «шолом» як мішень, стріляючи знизу вгору з землі. Крім того, спостереження анатомії «шолома» виявляють два різні типи відновлення тканин, що вказує на те, що кілька особин жили тривалий час після поранення. Дослідження підкреслює важливість конфіскованих зразків для розуміння особливостей життєвого циклу та впливу браконьєрства на рідкісні види, що є критично важливим для розробки цілеспрямованих стратегій збереження та поглиблення знань про біологію маловивченої дикої фауни.

## Traditional Chinese

盔犀鳥(*Rhinoplax vigil*)是一種極度瀕危的鳥類, 其頭顱喙部上方獨特的「盔突」使其備受覬覦, 如今正面臨被盜獵和棲息地喪失的雙重威脅。本研究將生物材料科學及影像技術應用於野生動物法醫鑑定領域, 運用微焦點電腦斷層掃描技術, 對香港漁農自然護理署(AFCD)查獲的一批珍貴盔犀鳥頭骨樣本, 展開槍彈造成損傷的法醫學分析。研究結果揭示了樣本呈現的多樣化創傷癒合反應、槍彈特徵, 以及可佐證盜獵者作案手法的相關線索。例如, 從頭骨組織中嵌有各式槍彈的樣本掃描影像可推斷, 盜獵者可能從地面向上射擊時, 將顯眼的盔突作為瞄準目標。此外, 透過對盔突解剖結構的觀察, 研究發現兩種不同類型的組織修復模式, 這表明部分樣本在遭受槍擊後仍存活了相當長一段時間。本研究顯出查獲的樣本對於探究珍稀物種的生活史要素與盜獵影響具有重要價值, 不僅為制定專屬保育策略提供關鍵依據, 亦有助於深化人們對這些難以追蹤的野生動物之生物學認知。

## Introduction

**Fig. 1:**
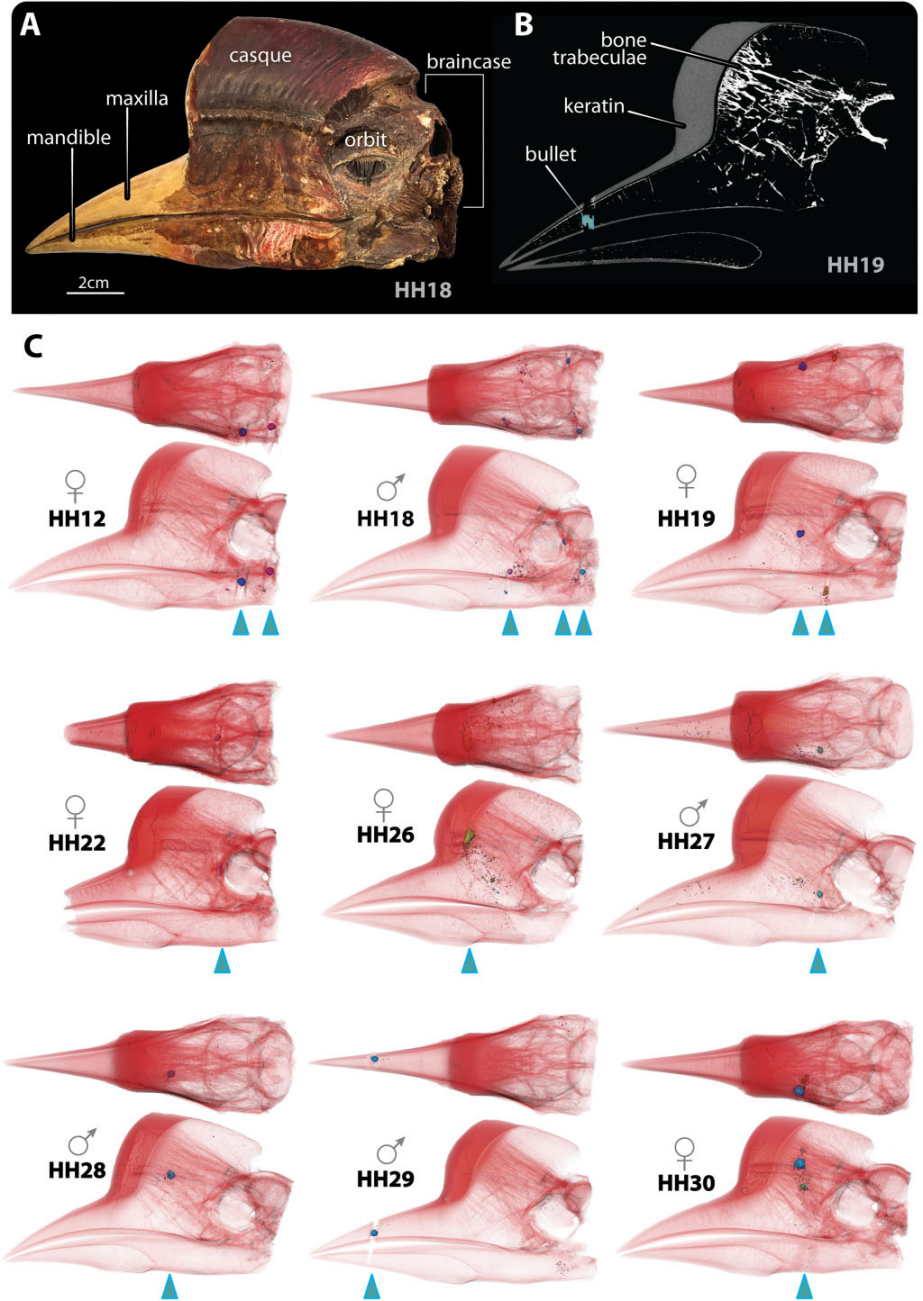
(A) External anatomical features of the helmeted hornbill skull. (B) Internal anatomical features of the casque; note the dense trabecular bone bridging the braincase and thickened keratin layer. (C) Thresholded volume renderings of the nine investigated specimens in dorsal and left lateral views, showing the location of primary bullets (blue arrowheads). Note, the defined linear streaks radiating symmetrically from some bullets (e.g., in HH12, HH18, HH19, HH27, HH29) are not bullet paths, but rather beam-hardening artefacts caused by the high density of the bullet; these artefacts could be easily distinguished from projectile tracks and secondary fracture lines in scan data.

The helmeted hornbill (*Rhinoplax vigil*) is a bird native to the Sundaic region of Southeast Asia (IUCN 2020). It differs from the other hornbills in the anatomy and appearance of its casque—a bright red cylindrical structure fronting the skull rostrally (Fig. 1A) (Manger Cats-Kuenen 1961; Kemp 2001). Unlike the casques of other hornbills where only a thin keratin shell confines a hollow cavity, the helmeted hornbill’s casque is reinforced by a massively thickened keratin layer enclosing a dense core of trabecular bone (Manger Cats-Kuenen 1961; Surapaneni et al. 2025) (Fig. 1B). This robust anatomical feature is believed to be associated with the bizarre aerial “jousting” behavior of this species, where individuals collide in mid-air (Kinnaird et al. 2003; Surapaneni et al. 2025; Schindler et al. 2025). Additionally, the substantial keratin layer of the casque makes it particularly appealing for ornamental carving, which has led to significant illegal poaching (Collar 2015). With the compounding effect of habitat loss, the helmeted hornbill is currently listed as “Critically Endangered” on the International Union for Conservation of Nature (IUCN) and in Appendix I of the Convention on International Trade in Endangered Species of Wild Fauna and Flora (CITES) (Beastall et al. 2016). Threats to helmeted hornbill populations also have broader implications for rainforest sustainability, as the species is a primary disperser of the seeds of strangler figs (Shanahan et al. 2001; Kinnaird and O’Brien 2008; Kitamura 2011), a “keystone species” for forest ecosystem structure (Chong et al. 2021).

Until two recent papers explored the internal architectures of the helmeted hornbill casque and intracranial joint (Surapaneni et al. 2025; Schindler et al. 2025), there was only a limited understanding of helmeted hornbill anatomy and physiology (e.g., Manger Cats-Kuenen 1961; Kinnaird et al. 2003). More information on the natural history and nature of population threats are needed to protect this endangered species and understand its role in the ecosystem. As poaching events are rarely observed, this study leveraged a unique specimen collection to perform a forensic investigation of the aftermath of helmeted hornbill poaching. Although not as common as human forensics, wildlife forensics is an advancing field and has already effectively characterized the ballistic injuries in different animal species (Zhang 2022). In this study, we take advantage of rare specimens to extend the utility of forensic investigations, providing novel information on the anatomy and physiological response to tissue injury in the helmeted hornbill casque and skull.

## Methods

Twenty dried helmeted hornbill skull specimens (Fig. S1) were collected from a single import confiscation by the Agriculture, Fisheries, and Conservation Department (AFCD) of the Hong Kong Special Administrative Region government in 2013. Specimens were donated to the City University of Hong Kong (CityU) for research purposes (AFCD ref: L/M 608/2022 in AF GR CON 07/13) and designated with CityU accession numbers; some of these specimens had been previously noted to contain remnants of bullets in their skulls (Webster 2022) and several others have been used in recent anatomical studies (Surapaneni et al. 2025; Schindler et al. 2025). Although skulls had been severed from their bodies by poachers at different locations post-cranially (Fig. S1), all 20 specimens possessed a part of the braincase, and both the bony (internal) and keratin (exterior) portions of the casque (Fig. 1A and B, Fig. S1). The sex of specimens was identified based on known sexual dimorphism, with females having black markings on the distal beak (Hatten et al. 2022).

### Preliminary scanning

We employed micro-computed tomography (μCT) for digital forensic imaging investigations of dried helmeted hornbill skulls. Considering the rarity of helmeted hornbill specimens, digital imaging is preferred over destructive necropsy for its ability to perform non-destructive internal structural investigation. Digital imaging approaches are also increasingly used in human forensic studies for reconstructing crime scene events (e.g., to study ballistic residues and trajectories) (Zhang 2022). Initial overview scans of the 20 specimens—to quickly identify specimens with bullets and fragments—were performed on a Comet Yxlon FF35 CT scanner at The Hong Kong Polytechnic University (100kV voltage, 35μA current, 1.5mm aluminium filter, 360° rotation, 1 projection per angular step, 9Hz frame rate, 100% detector sensitivity). Specimens with retained bullets and fragments were identified by examining scan projection images (Fig. S1). Among the 20 skulls, nine specimens (CityU ref: HH12, HH18, HH19, HH22, HH26, HH27, HH28, HH29, HH30) retained at least one primary (large) bullet piece; these specimens were selected as a subset for further detailed μCT forensic analysis (Fig. 1C, Fig. S1). Four specimens (HH9, HH15, HH21, HH23) from the original group of 20 animals possessed ballistic fragments, but retained no primary bullet (marked with asterisks in Fig. S1) and were excluded from additional analyses.

### Beam hardening artefact reduction

The primary goal of downstream forensic analyses was to examine the interaction of bullets and their fragments with bone tissue, requiring scanning parameters that allowed visualization of both organic and metal materials in the same scan. We used specimen HH22—one of the nine subset specimens, containing a bullet and fragments—to optimise scanning parameters for forensic analyses, to ensure scan quality and tissue discrimination and reduce beam hardening (metal scattering) artefacts in downstream detailed forensic analyses. We explored scanning parameters (Table 1) based on μCT studies of helmeted hornbill skulls lacking bullets (Surapaneni et al. 2025; Schindler et al. 2025), and on literature focused on beam hardening artefact reduction in µCT scans (Zhao et al. 2014; Huflage et al. 2022). Briefly, for most of the parameter sets we applied, we held constant beam voltages (200 kV), currents (30 µA), detector sensitivities (100%) and frame rates (9 Hz), while evaluating the effects of the number of projections taken (from 360 to 3600), filter presence/absence, and filters of differing materials (aluminum, copper, tin) and thicknesses (0.3–1.0 mm). Once those variables had been optimized, we adjusted current (30–140µA), detector sensitivity (25–100%) and frame rate (2.5–9Hz) for favorable images.

**Table 1:** Scanning parameters adjusted for beam hardening artefact reduction on μCT scan of HH22. Image quality is ranked on a five-point scale (1: worst, 5: best).

Parameter set	Voltage (kV)	Current (μA)	Projections	Filter	Filter thickness (mm)	Detector sensitivity (%)	Frame rate (Hz)	Image quality
1	200	30	360	None	N/A	100	9	1
2	200	30	360	Aluminium	0.5	100	9	1
3	200	30	360	Copper	1	100	9	1
4	200	30	360	Aluminium	1.5	100	9	1
5	200	30	360	Tin	0.3	100	9	1
6	200	30	1800	Aluminium	0.5	100	9	2
7	200	30	3600	Aluminium	0.5	100	9	3
8	200	30	3600	Copper	1	100	9	2
9	200	90	3600	Copper	1	100	9	4
10	200	90	3600	Tin	0.5	100	9	4
11	200	140	3600	Tin	0.5	100	9	4
12	200	140	3600	Tin	0.5	25	2.5	5

Reconstructed slices of the same anatomical location were compared from the scans across the range of tested parameters and evaluated by team members for quality (Table 1, last column; Fig. S2). The parameters that provided the optimal balance of bone tissue detail and minimal beam hardening artefact in reconstructed images involved comparatively high beam current (140 µA) and number of projections (3600), low frame rate (2.5 Hz) and detector sensitivity (25%), and a 0.5 mm tin filter (Parameter set 12; Table 1). These settings were applied for scanning the nine specimens in the subset group, for final resolutions of 72.3–79.1 µm (avg: 74.9 µm).

### Forensic analysis

Image reconstructions were performed by CERA (version 2206.4.0) using beam hardening correction with the primary material set to bone. The reconstructed images were rendered and evaluated using Amira software (ZIB Edition, v. 2022 Zuse Institute, Berlin). Bullet pieces were digitally segmented with automatic thresholding and manual selection. Areas/voxels where hyperdense photon starvation (streak) artefacts showed similar reconstructed greyscale values to the bullets were removed from the labelled field using a more stringent threshold window and manual correction.

**Fig. 2:**
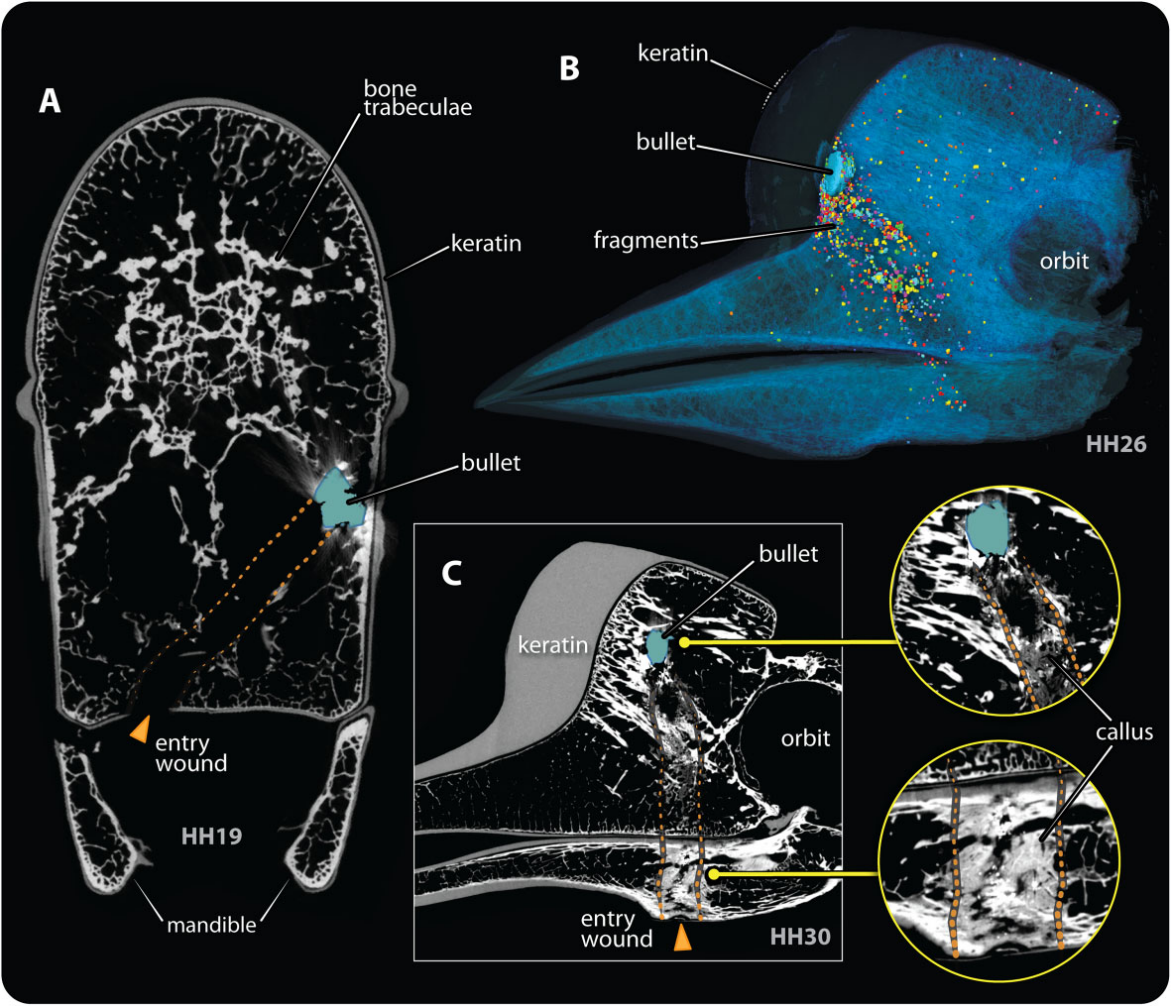
μCT scan images of specimens with bullets (pseudocolored blue), summarizing bullet appearance and types of skeletal damage observed in this study. (A) Coronal slice of HH19 showing the ballistic trajectory, evidenced by a path of bone fractures (orange dotted lines) connecting the entry wound (orange arrowhead) and the primary bullet. (B) Thresholded volume rendering of HH26, showing the primary bullet lodged between the keratin and bony portions of the casque with significant bullet fragmentation along the bullet’s entry path (individual fragments represented as different colors). (C) Sagittal slice of HH30 showing a partially mineralized callus filling the bullet’s entry path (orange dotted lines). Note that the thresholding in these images is adjusted to enhance the visualization of bone and keratin; this results in the bullets and their scatter appearing with similar grayscale values. During segmentation, a different threshold window was used to distinguish bullets from their scatter.

To analyse ballistic injury, we first observed physical specimens for entry fracture wounds and/or visible damage. The μCT images of each specimen were then examined in volume renderings and from multiple virtual sectioning planes to identify fractures internally. Since the interior of the bony portion of the helmeted hornbill’s casque is filled with a network of especially thick and highly aligned bony struts (trabecular bone; Doube et al. 2011; Surapaneni et al. 2025), fractures could be easily identified (Fig. 2). The density of the tissue associated with fracture regions was examined for signs of bone healing response: less radio-opaque material associated with a fracture zone was taken to indicate the presence of a soft callus (forming early during bone fracture repair; Espinosa et al. 2025), whereas a bullet encapsulated by tissue with radio-opacity similar to bone was interpreted as a fracture that had fully healed (Sabater González 2019).

The trajectories of bullets retained in skulls were identified using two methods. First, the Landmark Editor in Amira software was used to pinpoint tissue damage and fractures by moving slice-by-slice through the scans. This allowed us to build chains of landmarks that tracked tissue damage, connecting entry fracture wounds with the primary (largest) bullet piece. Once identified in µCT images, the locations of fracture wounds were verified on the physical specimens. Second, where no persistent fractures were evident, the spatial arrangement of bullet fragments was used to indicate the potential trajectory, as fragments tend to scatter conically, with the apex towards the entry direction (e.g., Fig. 2B) (Hanna et al. 2015). This method has been previously successfully demonstrated with the HH26 specimen (Webster 2022). All smaller elements (<10mm^3^) with radiodensity similar to the primary bullet were considered fragments.

Bullets and their fragments were analysed in Amira using qualitative and quantitative approaches. The shape of primary bullets was described from volume renderings, and their dimensions and volumes were measured using the Label Analysis module. The distance travelled by the primary bullets in the skull was calculated by summing the linear distances among successive landmarks defined in the Landmark Editor (see above). The number of fragments and the Euclidean scattering distance of all fragments from the primary bullet were also quantified from the Label Analysis output. Bullet volume retention (i.e., a common metric, the ratio of embedded primary bullet volume to intact bullet volume) was not calculated in this study because (1) fragments could not be accurately attributed to specific bullets in skulls with multiple bullets, (2) we could not be sure that all pieces of a bullet were retained in our cranial specimens and (3) any fragments retained in other body parts were not available to calculate the volume of the intact bullet. Quantitative bullet data were plotted and bullet size regressed against the number of bullet fragments using MATLAB (v. R2025b).

## Results

Of the 13 specimens out of 20 that retained bullet fragments, seven individuals (53.8%; Table 2) showed bullet fragments localized in the casque (i.e., the skull area filled with thick trabeculae; Figs. 1B, 2A and C), while the others showed bullets located either in the braincase, or the upper or lower beaks. Six out of nine specimens in the subset group were found to have only one primary bullet piece. In those with multiple large/primary bullet pieces, pieces were either co-located (HH12) or distributed broadly throughout the skull (HH18, HH19) (Table 2).

**Table 2:** Qualitative forensic analysis of 20 dried helmeted hornbill skull specimens. All specimens with primary bullets also contained fragments, see Fig. 4. The nine specimens with primary bullets used for further analyses are listed first (see also Table 3). With regard to bullet trajectory, a predominantly vertical orientation from the bird’s body toward its head is considered “caudocephalad,” while a horizontal trajectory in the direction from beak to cranium is “anteroposterior.”

ID	Sex	Bullet location	Fracture and tissue response	Bullet trajectory	Primary bullets i.e., pieces > 10 mm^3^
*Specimens included in further analyses (i.e., containing primary bullets, see Table 3)*					
HH12	F	Mandible	Complete bone healing	Not identifiable	2 round pieces
HH18	M	Mandible and maxilla on both sides, right orbit	Elevation and proliferation of keratin layer, bone fracture of right beak, complete bone healing on left beak	Not identifiable	2 round pieces + 1 deformed piece
HH19	F	Casque, right mandible	Persistent entry fracture on left palatine bone	Body-to-head (caudocephalad)	1 round piece + 1 deformed piece
HH22	F	Casque	Persistent entry fracture on midline palatine bone	Body-to-head (caudocephalad)	1 deformed piece
HH26	F	Casque	Persistent entry fracture on right maxilla	Body-to-head (caudocephalad)	1 deformed piece
HH27	M	Left maxilla	Complete healing	Beak-to-skull (anteroposterior)	1 round piece
HH28	M	Casque	Persistent entry fracture on left casque	Body-to-head (caudocephalad)	1 round piece
HH29	M	Cranial maxilla	Complete healing	Skull-to-beak (posteroanterior)	1 round piece
HH30	F	Casque	Soft callus formed, persistent entry fracture on left palatine bone	Body-to-head (caudocephalad)	1 deformed piece
*Specimens not included in further analyses (i.e., lacking primary bullets)*					
HH9	M	Braincase	No	N/A	Flecks only
HH15	F	Braincase	Complete bone healing	N/A	Flecks only
HH21	F	Casque, maxilla	Complete bone healing	N/A	Flecks only
HH23	M	Casque	Complete bone healing	N/A	Flecks only
HH11	F	N/A	N/A	N/A	N/A
HH13	M	N/A	N/A	N/A	N/A
HH14	M	N/A	N/A	N/A	N/A
HH16	F	N/A	N/A	N/A	N/A
HH20	F	N/A	N/A	N/A	N/A
HH24	M	N/A	N/A	N/A	N/A
HH25	M	N/A	N/A	N/A	N/A

### Tissue response

Different degrees of tissue healing were observed in the nine subset specimens (Figs. 1C, 2). Persistent entry wounds were observed externally in four of the specimens (HH19, HH22, HH26, HH28) (Fig. 2A and B). In HH12, HH27, and HH29, the primary bullets and fragments were completely embedded in casque trabecular bone, with the bone tissue encapsulating the bullet pieces having a similar density to the uninjured bone in the skull. In HH30, a mass (callus) of soft tissue opacity was observed connecting the entry wound and the primary bullet piece (Fig. 2C).

**Fig. 3:**
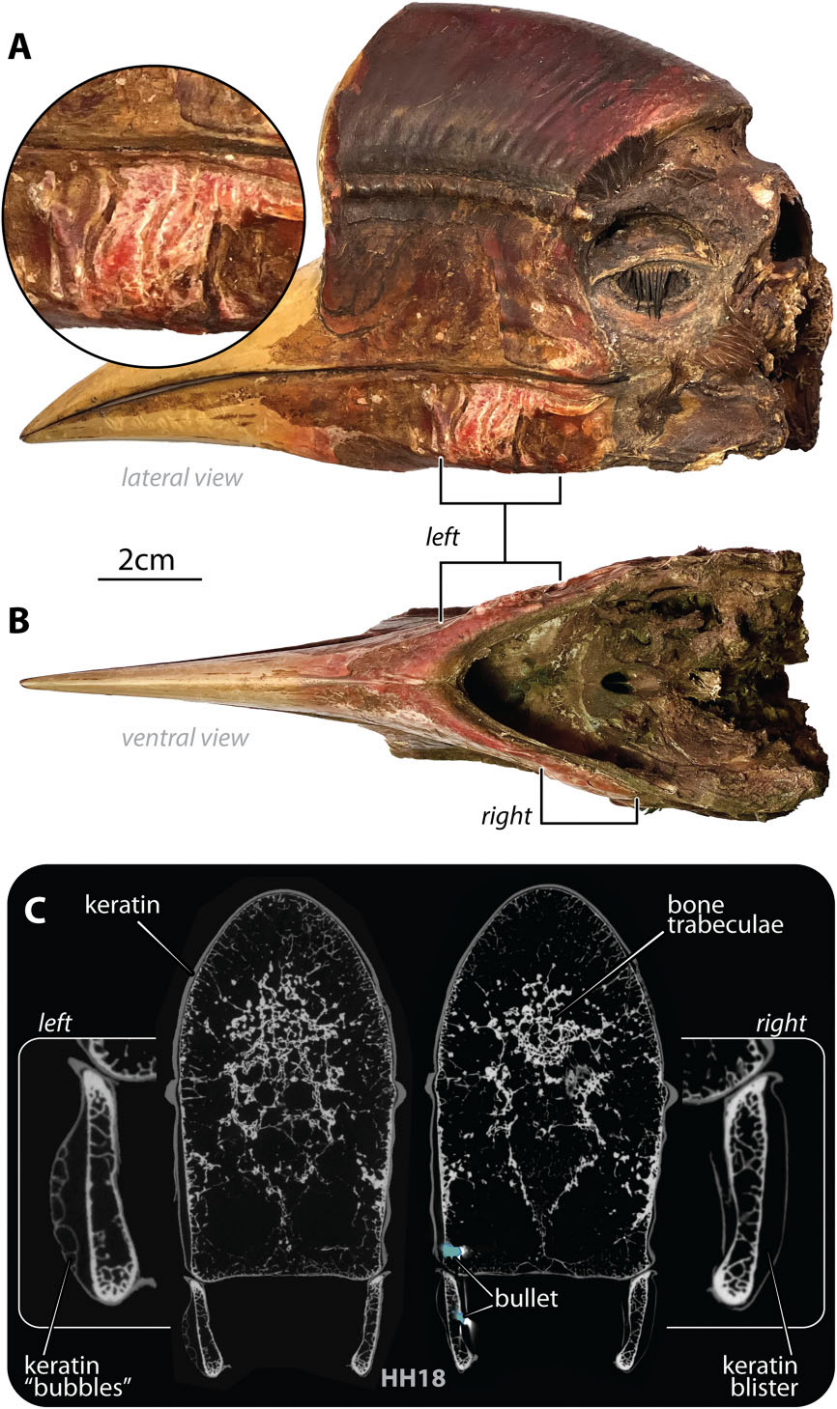
Keratin blisters observed on the mandible of specimen HH18. Photographs of the specimen in right lateral (A) and ventral (B) views show the swollen and discolored external appearance of the blisters on the left and right sides (indicated by brackets). Coronal sections through the blisters and mandible (C) reveal bubble microstructures in the left blister and a vacant blister on the right, with bullet fragments nearby (pseudocolored blue, as in Fig. 2).

In HH18, multiple bullet pieces were observed throughout the skull with differing degrees of persistent damage (Fig. 3). Whereas bone near bullet pieces on the left beak appeared undisrupted (i.e., healed), on the animal’s right side, fractures surrounded bullet fragments in the mandible and palatine bone. Close to the bullet entry locations on both sides of the mandible, the casque’s outer keratin layer exhibited visible “blisters” externally, where the keratin was elevated from the underlying bone (left: 22 × 12mm, right: 24 × 21mm, 22mm and 3mm away, respectively, from the germinal keratin edge at the caudal mandible margin, see Discussion) (Fig. 3). On the physical specimens these elevations were palpable and, in µCT, evident as large voids between keratin and bone, with the inner surface of the left blister exhibiting several smaller keratin “bubbles” (Fig. 3C). Bullet fragments embedded in the jaw were close to the blister on the left side, but not on the right (Fig. 3C).

### Trajectory analysis

In HH19, HH22, HH26, HH28 and HH30 persistent fracture paths could be used to locate the bullet entry wound and estimate ballistic trajectories (Fig. 2) (Hanna et al. 2015); as stated above, the bullet’s path in HH30 was filled by a non- or partially-calcified soft tissue (Fig. 2C). Secondary fractures were also observed near larger fragments in HH19, HH22, HH26. All trajectories identified in these five specimens were predominantly in the vertical (body-to-head) direction (e.g., Fig. 2B). In HH27 and HH29, no bone fracture was evident, as the bone around the bullets had completely healed; the spatial disposition of bullet fragments, however, suggested a predominantly horizontal bullet trajectory. In HH12 and HH18, trajectories could not be identified by either of our approaches (see Methods), as neither bone fracture paths nor conical fragment distributions were observed.

### Bullet analysis

**Fig. 4:**
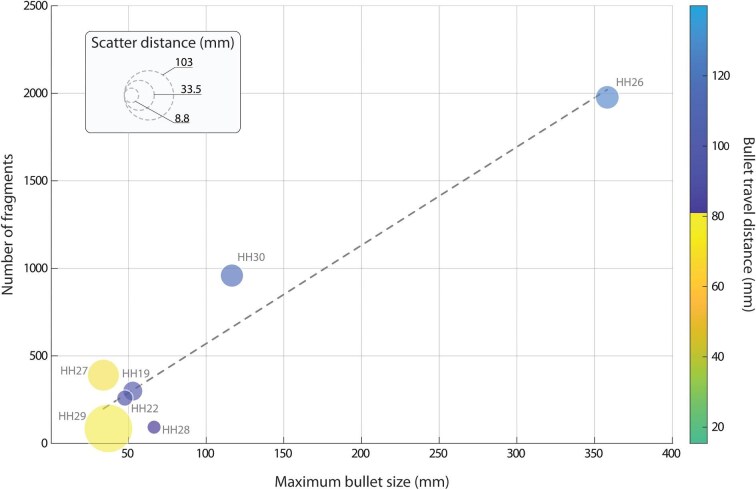
**Quantitative analysis of bullets in helmeted hornbill skulls.** There is a positive linear relationship between the maximum bullet size (x-axis) and the number of fragments (y-axis) separated from the primary bullets and distributed throughout the skull and casque. The color of each data circle represents the bullet travel distance (in mm; second y-axis), while circle diameter indicates the scatter distance of detected fragments (key: upper left corner). Specimens HH26 and HH30 retained the highest number of fragments, while the others contained fewer than 500. HH12 and HH18 are not included, as their multiple primary bullets complicated the calculation of individual trajectories and fragment attribution (see Table 3). Regression: (y = 5.62x + 8.26), p = 0.000656, r^2^ = 0.92.

A high variation of bullet characteristics was observed among subset specimens (Fig. 4, Table 3). The primary bullet pieces in six specimens were spherical, ranging in volume from 25mm^3^ to 66mm^3^. The bullets in the other three specimens (HH22, HH26, HH30) were deformed and the original shape could not be reconstructed (e.g., Fig. 2B). Two of these deformed bullets were considerably larger than the spherical bullets (HH26: 358.2mm^3^; HH30: 116.6mm^3^). The remaining deformed bullet in HH22 (47.8mm^3^) had a similar volume to the spherical bullets. An outstandingly high number of bullet fragments was observed in HH26 (1977 pieces; Fig. 2B) and HH30 (959 pieces), while all other specimens had < 500 fragments. In specimens where trajectories were identifiable, bullets had travelled on average 60.40 ± 50.43 (maximum: 139.9mm) in the skull. A positive linear relationship was observed between the size of the primary bullet and the number of fragments retained (regression: y = 5.62x + 8.26; p = 0.000656; r^2^ = 0.92; Fig. 4, Table 3).

**Table 3:** Quantitative forensic analysis of the nine dried helmeted hornbill skull subset specimens. Fragment trajectories and scattering distance could not be calculated for specimens with multiple primary bullet pieces (those shot by multiple bullets, i.e., HH12, HH18, HH19). In HH19, one larger bullet was observed in the casque and a smaller one in the mandible. The mandibular piece was considerably larger than most fragments we observed, but smaller than most primary bullets; from this and the surrounding damage, we could not determine whether it was a fragment of the casque bullet or rather a primary bullet in its own right. This piece and closely associated fragments were therefore excluded from scattering/trajectory analysis. In contrast, the casque bullet showed a clear entry trajectory and associated fragmentation.

ID	Primary bullet volume (mm^3^)	Number of fragments	Trajectory distance (mm)	Scattering distance (mm) [means ± stdev; maximum]	Median scattering distance (mm)
HH12	51.8, 63.9	81	N/A	N/A	N/A
HH18	19.8, 25.1, 26.0	465	N/A	N/A	N/A
HH19	52.8 (casque) 15.9 (mandible)	299 (171, excluding mandible fragments)	33.97	17.66 ± 11.53; max 72.05	14.52
HH22	47.7	259	27.8	11.58 ± 9.05; max 43.86	8.01
HH26	358.2	1977	54.92	24.75 ± 16.32; max 78.70	20.50
HH27	33.9	390	131.21	44.91 ± 29.37; max 131.21	33.57
HH28	66.5	93	15.35	8.78 ± 8.99; max 52.36	5.60
HH29	37.1	86	139.95	102.98 ± 53.84; max 139.95	131.94
HH30	116.6	959	46.07	23.84 ± 17.47; max 68.47	18.34

## Discussion

### Tissue healing response and survival time

Diverse healing phases of the helmeted hornbill casque were opportunistically captured in this study, suggesting timelines for poaching events. The bone healing process in avian species is similar to that in mammals (Tully 2002; Sabater González 2019). Initially, fracture gaps are bridged with fibrous connective tissue and cartilage (i.e., soft callus) produced by the periosteal cells, endosteal cells, and fibroblasts near the fracture site (Oryan et al. 2015; Sabater González 2019). This early stage of bone healing is evident in HH30, where the fracture gap is filled with materials of soft-tissue density (Fig. 2C). This is perhaps the first evidence of casque repair in the helmeted hornbill and indicates that HH30 had likely survived for probably up to three weeks after being shot, as soft callus formation is typically seen 9–21 days post-injury in birds (Sabater González 2019).

In contrast, the sharp fracture edge with uniform tissue density (i.e., lacking calluses) in four specimens (HH19, HH22, HH26 and HH28) suggests that those birds did not survive long enough to deposit calluses into the fracture gap. From literature recording casque surgeries in hornbills under rehabilitative care, it has been seen that the casque in other species is not extremely vascularised (Wright 1992; Xie et al. 2019), suggesting that ballistic damage to the casque itself would likely not prove fatal. However, the degree of vascularization of the helmeted hornbill’s impressive casque has not been adequately described and clearly possesses considerably more bone (a vascular tissue) than the casques of other hornbills (Schindler et al. 2025). We posit that the birds in our study were knocked out of trees by the shot and collected by poachers within a fairly short time.

In specimens where bullet-associated damage appears completely healed, it is more difficult to estimate post-injury survival time. In other species, mineralisation of fracture calluses typically occurs 4–12 weeks post-injury (Tully 2002; Sabater González 2019). The actual healing time of individuals in this study may have even been longer, due to the lack of proper treatment for these wild-caught birds (Sabater González 2019). As the remaining body parts were not examined in this study, the actual cause of death could not be confirmed. The apparently long post-injury survival times of some individuals with extensive bullet wounds argue that the casque is not vital for survival. However, casque damage and subsequent infections in other hornbills have led to neurological defects (Wright 1992; Gjeltema et al. 2015), implying that casque injury in the helmeted hornbill could indirectly hinder foraging ability, eventually leading to easy capture by poachers. Exposure to lead has been shown to inhibit fracture healing, so our estimations of the age of injuries based on the stage of fracture healing may be affected by the presence of lead in the bullets (Carmouche et al. 2005) (see discussion of bullet materials below). There is no easy way to correct for this, however, as a great number of fractures can increase the systemic levels of lead from retained bullets in cranio-maxillofacial regions, resulting in a complex causality (Edetanlen and Saheeb 2019).

The specimen with keratin “blisters” (HH18; Fig. 3) further indicates casque repair. In birds with simpler beak structure (i.e., lacking casques), keratin grows continuously from the germinal keratin edge at the caudal margin of the beak to replace older keratin distally (Huynh et al. 2019). Although the speed of keratin growth varies among species (Wu et al. 2006), beak keratin is believed to grow slowly (e.g., ∼0.25 mm/year in adult toucans, with mandibular keratin growing particularly slowly; Emily and Eisner 2021). Indeed, keratin was shown to only begin production from granulation tissue 5 months after casque damage in an injured northern pied hornbill (*Anthracoceros albirostris*) (Wright 1992). Information on helmeted hornbill beak keratin growth and repair is lacking, but data from other bird species indicate that more time is required to heal keratin than bone. If the caudal-to-rostral mechanism of keratin growth of other birds holds for helmeted hornbills, the locations of blisters in HH18 relative to injury sites suggests that the tissue insults likely occurred some time before death (i.e., repaired keratin areas had time to shift rostrally). Furthermore, the bubbling microstructure in the left mandible’s keratin blisters may indicate that it was formed in a manner or in response to a type of damage different from the right blister (lacking bubbles; Fig. 3C). The µCT images shown here may be the first high-resolution documentation of casque healing: the blister architecture and the variable bubbling microstructures observed require deeper investigation to understand keratin healing in hornbills and also its effect on beak integrity.

### Poaching strategies

In more than half of the 20 investigated specimens, bullets were localized in the casque, which suggests that this brightly coloured structure may be a common target for poachers. We acknowledge that our modest sample size and the lack of information on the poaching locale (not known by the AFCD) may represent a sampling and/or survival bias, as the birds may have come from a single region and/or trader. However, given the low population density of helmeted hornbills (Collar 2015), we posit that our samples likely capture a relatively wide geographic area and therefore an important snapshot of poacher-hornbill interactions. Individuals having been targeted multiple times are also indicative of the rarity of this species and perhaps individual poachers hunting in relatively limited regions. In contrast, the lack of wound healing in some specimens argues that the observed poaching event was the only one those birds encountered, with a single shot being enough to critically injure the bird.

As the helmeted hornbill is an arboreal species (Kemp 2001), the dominant vertical (body-to-head) bullet trajectories observed in many specimens suggest poachers likely fired bullets from the ground. In contrast, for the two specimens (HH27, HH29) with horizontal bullet trajectories, poachers may have shot the birds from a certain height (e.g., an adjacent tree). As both birds are male, those poaching events might have happened when the individuals stopped by the nest to feed the female and chick, which are sealed together in a hole within a tall forest tree during the 5–6 month nesting period. As the female and chick rely entirely on the male for resources during this time (Kaur et al. 2018; Hatten et al. 2022), it could be an effective strategy for poachers to locate the nest, scale a nearby tree, and wait for the male. Although a complete healing response was observed in these two males, in general, damage to males during the nesting season is especially dangerous for the mate pair, as it could seriously hinder foraging ability and thereby significantly lower survival chances for females and chicks (Hatten et al. 2022). It is more difficult for poachers to reach females when they seal themselves in the nest (Kaur et al. 2018); the female specimens in our sample therefore were likely poached outside the nesting season, indicating that poaching events targeting the helmeted hornbill may occur throughout the year.

### Poaching equipment

A variety of bullet characteristics were identified in our specimen subset. In three specimens, retained bullets were heavily deformed, indicating that they are likely lead, in soft- or hollow-point structure and without a metal jacket (Thomas 2013; Hanna et al. 2015). This aligns with our expectations, as lead bullets are a common, low-cost choice for hunters (Caudell et al. 2012; Thomas 2013), whereas bullets with a well-retained spherical shape are more likely to be made of metals harder than lead (Hollerman et al. 1990). The fragmentation behaviour we observed also supports this hypothesized difference in bullet material (Fig. 4, Table 3). Non-lead bullets fragment less and give much larger fragments than lead bullets (Grund et al. 2010; Hampton et al. 2021; McTee et al. 2023). Indeed, the number of fragments in specimens shot by spherical (i.e., probably non-lead) bullets was far lower than that from deformed bullets (Fig. 4, Table 3). It is worth noting that the number of fragments may even be underestimated in our datasets, as beam hardening artefacts can obscure bullet-adjacent fragments, and extremely small fragments (i.e., smaller than our scan resolution) could have been missed.

Beyond bullet material and construction (Oura et al. 2024), fragmentation intensity is known to depend strongly on the amount of energy transferred when a projectile deforms (Penn-Barwell et al. 2015). Although few peer-reviewed studies directly assess the influence of bullet size on fragmentation, experimental work on bullets of similar design suggests that heavier projectiles shed substantially more fragments (Grund et al. 2010; Hampton et al. 2021; McTee et al. 2023). Additionally, impacts with rigid tissues, such as bone or keratin (as in the hornbill’s casque), induce particularly extensive fragmentation (Caister et al. 2020, Oura et al. 2024). The relationships among bullet characteristics observed in our datasets (Fig. 4, Table 3) are therefore consistent with basic wound-ballistic principles: larger bullets of otherwise similar construction carry more kinetic energy and deformable mass, leading to increased expansion and secondary fragmentation, especially upon bone impact. In this regard, the small size of the observed bullet and low number of fragments in HH22 contrasts with other deformed bullets, suggesting that the actual primary bullet may have been either lost or retained in another body part.

## Conclusions

Our exploration of poaching-associated helmeted hornbill cranial injury in rare skull specimens frames novel concepts in avian tissue biology that are impossible to explore in living animals, while underlining the deep value of specimen collections for informing natural history and conservation biology. Accessible specimens of the helmeted hornbill are exceptionally scarce worldwide: this study shows how the utility of even confiscated specimens can be maximized for understanding human-wildlife interactions and crafting targeted conservation measures for endangered species. Using high-resolution μCT, we additionally revealed detailed ballistic signatures—including bullet deformation, fragmentation fields, and scatter patterns—that would otherwise be unobserved. These data create opportunities for systematic comparisons of projectile behaviour across biological tissues of specimens and highlight μCT’s potential as a forensic tool for wildlife crime. The need to rapidly identify bullet materials (e.g., for medical and conservation forensics), coupled with the current lack of reference Dual Energy Index (DEI) measurements for metals commonly used for bullet manufacturing (Gascho et al. 2020), poses a demand for more study and standardized methods for bullet characterization. In our dataset, the strong linear relationship observed between primary bullet size and number of fragments is clearly heavily influenced by the two largest bullets (Fig. 4); exploring and fleshing out this interaction in future studies would be valuable for diverse forensic fields. Paired with the distinct and reproducible fragmentation signatures from bullets of different materials, a more robust suite of bullet identification tools would be particularly powerful for wildlife forensics (e.g., for characterizing specific poaching equipment even when the primary projectile is deformed or absent).

## Supplementary Material

obag001_Supplemental_Files

## Data Availability

Lateral projections from µCT scans from all specimens studied and a spreadsheet of quantitative ballistic measurements from segmented scan data are available via Figshare: https://figshare.com/projects/A_material_approach_to_endangered_species_conservation_Characterization_and_3D_imaging_of_ballistic_damage_in_helmeted_hornbill_Rhinoplax_vigil_casques/247265
