# Supplementary figures and images for: A Material Approach to Endangered Species Conservation: Characterization and 3D Imaging of Ballistic Damage in the Casques of Helmeted Hornbill (Rhinoplax vigil)
Source: Integr Org Biol. 2026 Jan 19;8(1):obag001. doi: 10.1093/iob/obag001 (PMC13100508; doi:10.1093/iob/obag001)

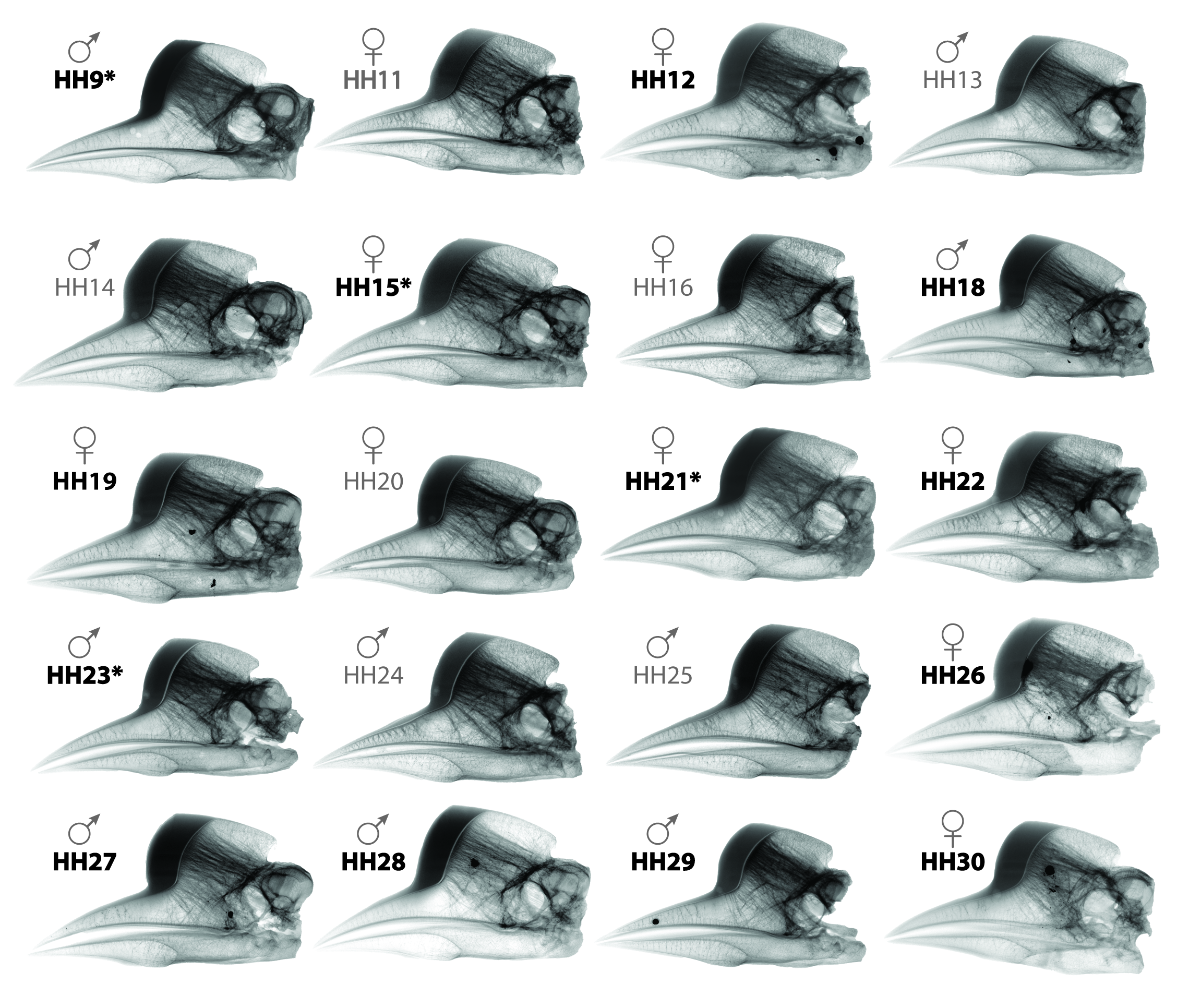

Supplement: obag001_Supplemental_Files [file obag001_supplemental_files.zip › FIGS_Hornbill-bullets-FigS1.tif]

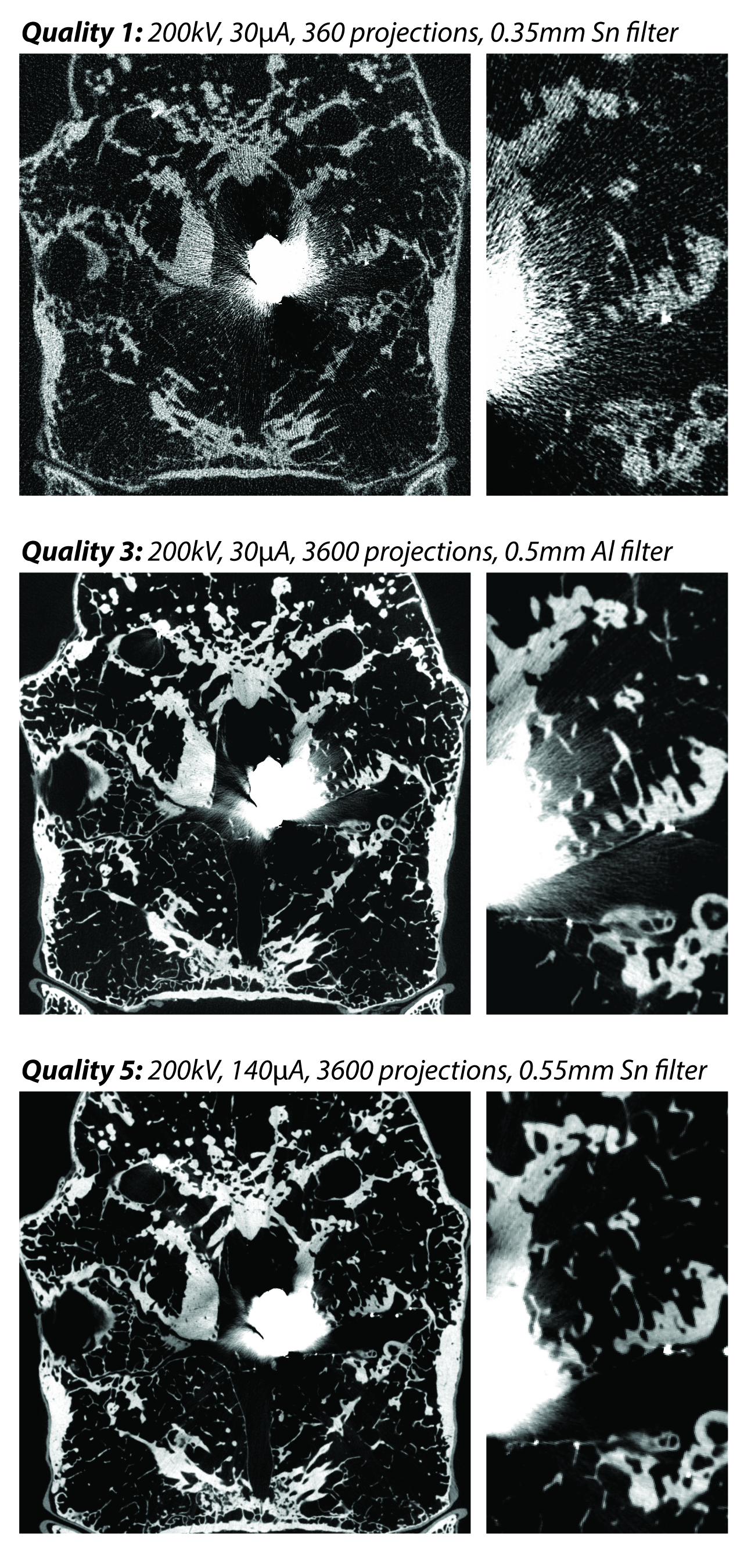

Supplement: obag001_Supplemental_Files [file obag001_supplemental_files.zip › FIGS_Hornbill-bullets-FigS2.tif]
